# Differential effects of emotional cues on components of prospective memory: an ERP study

**DOI:** 10.3389/fnhum.2015.00010

**Published:** 2015-01-28

**Authors:** Giorgia Cona, Matthias Kliegel, Patrizia S. Bisiacchi

**Affiliations:** ^1^Department of General Psychology, University of PaduaPadua, Italy; ^2^Department of Psychology, University of GenevaGenève, Switzerland; ^3^Department of General Psychology, Center for Cognitive Neuroscience, University of PaduaPadua, Italy

**Keywords:** prospective memory, emotion, ERPs, delayed intentions, LPP, event-related potentials, PLS, neural

## Abstract

So far, little is known about the neurocognitive mechanisms associated with emotion effects on prospective memory (PM) performance. Thus, this study aimed at disentangling possible mechanisms for the effects of emotional valence of PM cues on the distinct phases composing PM by investigating event-related potentials (ERPs). Participants were engaged in an ongoing N-back task while being required to perform a PM task. The emotional valence of both the ongoing pictures and the PM cues was manipulated (pleasant, neutral, unpleasant). ERPs were recorded during the PM phases, such as encoding, maintenance, and retrieval of the intention. A recognition task including PM cues and ongoing stimuli was also performed at the end of the sessions. ERP results suggest that emotional PM cues not only trigger an automatic, bottom-up, capture of attention, but also boost a greater allocation of top-down processes. These processes seem to be recruited to hold attention toward the emotional stimuli and to retrieve the intention from memory, likely because of the motivational significance of the emotional stimuli. Moreover, pleasant PM cues seemed to modulate especially the prospective component, as revealed by changes in the amplitude of the ERP correlates of strategic monitoring as a function of the relevance of the valence for the PM task. Unpleasant pictures seemed to modulate especially the retrospective component, as revealed by the largest old/new effect being elicited by unpleasant PM pictures in the recognition task.

“*A sight, an emotion, creates this wave in the mind, long before it makes words to fit it*.”

*Virginia Woolf*

## Introduction

### Prospective memory and emotions

Remembering to execute delayed intentions at the appropriate time in the future is classically referred to as Prospective Memory (PM; Brandimonte et al., 1996). PM intentions are ubiquitous in our everyday life, and successful accomplishment of intentions has important implications on health (e.g., remembering to take medications), social life (e.g., returning a book to a friend), business (e.g., submitting a paper before deadline), and safety (e.g., remembering to switch off the stove after cooking).

PM is a complex function and is composed of multiple components, phases and processes (e.g., McDaniel and Einstein, 2000; Kliegel et al., 2002). More specifically, PM entails a retrospective and a prospective component. The retrospective component supports recalling of the content of the intention from memory, whereas the prospective component involves retrieving the intention upon encountering the appropriate event, which is typically called “PM cue” (Einstein et al., 1992). PM can also be conceptualized as composed of four main phases: encoding of an intention, intention maintenance, intention retrieval and intention execution (Kliegel et al., 2002, 2011).

Finally, PM may require a plurality of processes, as postulated by the Multiprocess view (McDaniel and Einstein, 2000, 2007; Scullin et al., 2013). Here, it has been proposed that, under some circumstances, PM is mediated by strategic monitoring, which consists of a set of attentional and memory processes recruited to monitor the environment for the occurrence of the PM cue (i.e., target checking) and to maintain the intention active in mind (i.e., retrieval mode) (McDaniel and Einstein, 2000; Guynn, 2003; Cona et al., 2012a,b). Under different circumstances, the intention can be spontaneously retrieved without the allocation of strategic resources (Moscovitch, 1994; McDaniel and Einstein, 2000). Multiple factors were found to modulate the extent to which a PM task invokes strategic monitoring processes, such as focality and perceptual salience of the PM cue (McDaniel and Einstein, 2000; Cona et al., 2014). Among these factors, emotional valence could be another important variable that modulates PM process components, given that emotional valence of the information was shown to have a powerful influence on, for example, episodic memory or attention (Murphy and Isaacowitz, 2008). So far, however, empirical findings on the effects of emotions on PM are still sparse and inconclusive (Kliegel and Jäger, 2006; Clark-Foos et al., 2009; Altgassen et al., 2010; Rendell et al., 2011; May et al., 2012, 2014; Rummel et al., 2012; Schnitzspahn et al., 2012). Only few studies investigated the influence of PM cue valence in younger adults' performance, showing partially opposite results. Altgassen et al. (2010) reported that emotional valence did not have a significant impact on PM performance in young individuals. Clark-Foos et al. (2009) found instead that unpleasant PM cues triggered prospective intentions less frequently compared to pleasant PM cues. The authors interpreted this result suggesting that unpleasant PM cues automatically trigger unpleasant thoughts that act as an additional task interfering with PM performance. Nevertheless, they did not include a neutral condition, thus it was not possible to ascertain whether either unpleasant or pleasant PM cues would have produced a performance advantage/disadvantage compared to neutral PM cues. Rendell et al. (2011) added a neutral condition and observed better PM performance with pleasant cues than with unpleasant and neutral cues, thus revealing a positivity-related enhancement but not a negativity-related change in performance. By contrast, the studies by May et al. (2012, 2014) showed that pleasant and unpleasant PM cues may lead to a similarly higher level of PM performance as compared to neutral PM cues. The enhanced PM performance with emotional cues was not the result of greater attention or effort to the PM task as there were not increased costs on the ongoing task when emotional PM tasks, rather than neutral PM task, were added. These data were interpreted as indicating the efficacy of emotion in boosting cue saliency, reducing the need for strategic monitoring. Also, the equivalent advantage for pleasant and unpleasant cues seemed to be due to the arousing nature of these stimuli, which made them more salient compared to non-emotional ongoing stimuli.

Taken together, results of available studies are heterogeneous leaving the impact of emotional valence on PM performance an open question. Moreover, the *mechanisms* of possible emotion effects are even more poorly understood as none of the previous studies has clearly investigated which component, phase, and process of the PM may be especially sensitive to the effect of emotional valence of the PM cue. Hence, in the present study, we decided to use event-related potentials (ERPs) to further explore those questions. ERPs are a powerful tool to provide information about the effects of valence on distinct and specific cognitive processes underlying PM and they can yield conceptually important insights also for those PM phases, such as, for example, the encoding phase, which do not require any behavioral response.

### ERPs as a tool to study the interplay between PM and emotions

Regarding ERP correlates of PM, two main classes of ERPs are usually analyzed: ERPs elicited by ongoing trials in PM blocks, and ERPs elicited by PM cues themselves. Specific ERPs elicited by ongoing trials (in blocks where PM instructions are also given) have been suggested as electrophysiological markers of strategic monitoring by us and other labs: (West et al., 2007; Knight et al., 2010; Czernochowski et al., 2012; Cona et al., 2012a,b, 2014). These are typically expressed as sustained positive modulations starting roughly at 200–300 ms and lasting for several 100 ms. These modulations are widely distributed over the scalp but particularly pronounced over frontal and prefrontal regions (e.g., Knight et al., 2010; West et al., 2011; Czernochowski et al., 2012; Cona et al., 2012a,b, 2014). Transient frontal and parietal modulations were also shown, with a timing varying as a function of the PM cue defining features, and were considered to be mainly related to target checking (West et al., 2007; Knight et al., 2010; Cona et al., 2012a, 2014; Scolaro et al., 2014).

The ERPs elicited by PM cues provide information about the processes underlying the detection of the PM cue as well as the retrieval and execution of the delayed intention when the PM cue is encountered. The N300 is a negative deflection, occurring in the 300–500 ms time window over the occipital–parietal regions of the scalp and is related to the detection of the PM cue in the environment (e.g., West and Ross-Munroe, 2002; West and Krompinger, 2005; West, 2007; Zöllig et al., 2007; Bisiacchi et al., 2009). The parietal positivity is the result of three multiple overlapping components: the P300, the old/new effect and the prospective positivity (West et al., 2001; West and Ross-Munroe, 2002; West and Krompinger, 2005; West, 2011). This component is expressed over parietal and centroparietal regions of the scalp between 400 and 1200 ms after stimulus onset and is related to memory processes, such as PM cue recognition and intention retrieval (West et al., 2001; Zöllig et al., 2007; Bisiacchi et al., 2009; West, 2011; Cona et al., 2014).

Importantly for our purposes, there is ample evidence that several ERP components are sensitive to the emotional valence of stimuli in cognitive tasks in general. Emotion-related modulations of early components of the ERPs (e.g., P1, N1, and P2) have been observed repeatedly and were thought to mainly reflect the automatic capture of attention by emotional stimuli (possibly because of the evolutionary significance of emotional information, see e.g., Carretiè et al., 2004). Two other ERP components are classically explored in the field of emotion neuroscience: The Early Posterior Negativity (EPN) and the Late Positive Potential (LPP). The EPN is a relative negative deflection elicited by emotional stimuli over occipital sites around 200–300 ms post-stimulus, and is associated with an increase in the allocation of attentional and processing resources toward emotional stimuli (particularly pleasant stimuli) compared to neutral stimuli (e.g., Schupp et al., 2006; Foti et al., 2009; Weinberg and Hajcak, 2010). The LPP is a sustained positivity that begins around 300 ms after stimulus presentation and lasts for 1000–2000 ms (e.g., Foti and Hajcak, 2008; Weinberg and Hajcak, 2010). It is mostly expressed over central and parietal regions of the scalp and is greater for both pleasant and unpleasant pictures than for neutral pictures (e.g., Cacioppo et al., 1993; Schupp et al., 2003; Olofsson et al., 2008; Foti et al., 2009). The influence of valence on the LPP amplitude has been shown to interact with the relevance of the emotional content for task performance, being greater when the emotion was relevant for the goals of the task. This finding suggests that the LPP is susceptible to top-down processing and motivational influences (Hajcak et al., 2006) and reflects allocation and holding of attention toward emotional stimuli, especially when they have motivational relevance (Schupp et al., 2004; Hajcak et al., 2006, 2009; Olofsson et al., 2008).

### The current study

The current study, for the first time, applies an ERP approach to the study of emotion effects on PM. Specifically, it was designed to disentangle possible mechanisms of emotion effects on PM performance across the process phases of prospective remembering and also including a test of retrospective memory for the content of the PM task.

Considering the inconclusive pattern revealed by the available literature, a number of different possible hypotheses were explored:

As summarized above, according to the Multiprocess view (McDaniel and Einstein, 2000, 2007), accomplishing a PM intention can be mediated by a number of distinct processes, ranging from strategic monitoring processes to more spontaneous retrieval processes. Emotions are discussed to increase the saliency of the PM cue (Altgassen et al., 2010; May et al., 2012), so they could reduce the need for strategic resources for PM cue detection, which would then rely more on the automatic capture of attention. If this was true, one would expect effects of emotion on the earlier ERP components related to PM, such as the N300, which is sensitive to attentional resources recruited for PM cue detection (West et al., 2003; West, 2011). Emotions could also have an impact on the retrospective component, for example boosting the spontaneous retrieval of intention (Rendell et al., 2011). If this was true, this should be observed in a modulation of the FN400, which reflects automatic retrospective processes (Curran, 2000; West, 2011).

On the other hand, emotional PM cues may encourage strategic monitoring given that emotional information can increase the relevance of the PM task, enhancing motivational processes supporting PM performance (Kvavilashvili and Fisher, 2007; Aberle et al., 2010). Based on this argument, one would expect to observe an increased positivity of the sustained ERP modulations associated with strategic monitoring in the ongoing trials (e.g., Czernochowski et al., 2012; Cona et al., 2012a, 2014). It is also plausible that enhanced motivation for emotional PM tasks is accompanied by an increase in the LPP—an ERP component sensitive to motivational factors—elicited by the emotional stimuli which might show up during all PM phases (Schupp et al., 2000, 2003; Hajcak et al., 2009).

Further assumptions have been made in relation to the role of emotions for the encoding of an intention (see Kvavilashvili and Ellis, 1996, for initial ideas); if the encoding phase was indeed the route through which emotional PM intentions are better executed, then one would predict effects of emotions already on the ERPs elicited during this phase.

## Methods

### Participants

Twenty-four students from the University of Padua (10 males, *M* = 23.5 years, range = 20–28 years) took part in the study for partial course credits. For the determination of the sample size we used G^*^Power (Faul et al., 2007). To detect a medium effect size (0.25), with power (*1– β*) set at 0.80 and α = 0.05, the recommended minimum sample size comprised 15 participants. This estimation was done in relation to the behavioral analysis on ongoing task. However, since in the present study we focused on ERP analyses, we decided to recruit a larger sample size of participants (*N* = 24) following one of our previous PM studies that obtained reliable ERP findings by using the same ERP approach (i.e., Partial Least Square analysis; Cona et al., 2014).

All participants had normal, or corrected-to-normal, vision and no neurological or psychiatric pathologies. Participants reported themselves to be in good physical and mental health. Given the study material, it was screened for particular phobias as exclusion criterion (e.g., snake phobia, blood phobia). None of the participants reported to have phobic fears, so they have been all recruited in the study.

The study was approved by the ethical committee of the School of Psychology of the University of Padua and was conducted according to the principles of the Declaration of Helsinki. All the participants were informed about the general procedure of the experiment and signed a written consent form.

### Material

The PM task was designed after previous behavioral studies on emotion and PM (e.g., Altgassen et al., 2010). Specifically, 228 pictures were selected from the International Affective Picture System IAPS (Lang et al., 2005). Seventy-six were pleasant, 76 neutral, and 76 unpleasant pictures. These picture types varied significantly in IAPS normative valence ratings, with lowest valence for unpleasant pictures (*M* = 2.8, *SD* = 0.6), intermediate valence for neutral pictures (*M* = 5.0, *SD* = 0.2), and highest valence for pleasant pictures (*M* = 7.3, *SD* = 0.6). In terms of emotional arousal, pleasant and unpleasant pictures did not differ from each other (pleasant: *M* = 5.1, *SD* = 0.6; unpleasant: *M* = 5.2, *SD* = 0.4; *p* > 0.05), whereas as usually in this type of study both pleasant and unpleasant pictures differed from neutral pictures (neutral: *M* = 3.3, *SD* = 0.6, *ps* < 0.05).

Pictures were chosen from four categories: persons, animals, landscape, and objects, with a balanced number of pictures per valence. More specifically, within the overall category of unpleasant valence, images of illness (e.g., a sick man), of blood, drug abuse (e.g., a syringe), images of threat (e.g., a gun), disgusting images (e.g., an overflowing toilet), images of dead or disgusting animals, and images of threatening weather phenomena (e.g., a tornado) were selected. Within the overall category of neutral valence, images of landscapes (e.g., buildings), images of common household tools/objects (e.g., a spoon), of food and mushrooms, scenes with people (e.g., an old man with neutral face expression), were selected. Within the overall category of pleasant valence, exciting images (e.g., exciting sports), delicious food, erotic scenes, affiliative pictures (e.g., babies, cuddly animals, smiling families), and exotic/peaceful landscapes were selected. We decided to select images from a wide range of semantic categories in order to attenuate potential effects of one category (e.g., erotic images) on the other ones. For each valence type, 25 pictures were selected as PM cues and the remaining pictures as ongoing stimuli. PM and ongoing stimuli were matched for valence and arousal level. Pictures were presented in the center of a black screen.

### Procedure

Following previous paradigms (e.g., Einstein et al., 1992; Altgassen et al., 2010), each PM session was comprised of an ongoing working memory task and a PM task that consisted in remembering to make a prospective key-press whenever a specific picture occurred.

The ongoing task was a one-back working memory task entailing neutral, pleasant, and unpleasant pictures. Participants were required to decide whether the picture occurring on the screen was the same or different from the picture occurring one trial before and to indicate their decision by pressing one of two possible response keys with the index or middle finger of their right hand (“N” or “M” keys on the keyboard). The response-key mapping was counterbalanced across participants. On each trial, the stimulus remained on the screen for 2000 ms or until a response was made, followed by a black screen with a fixation cross that pseudorandomly lasted 1200, 1400, or 1600 ms.

The experiment was composed of three PM sessions, which varied for the emotional valence of the PM cue (pleasant, unpleasant, neutral). Participants had to remember to press the prospective memory key (i.e., “Z” key) with their left index finger, after having made the one-back response, whenever a PM cue was presented. Within a PM session, pleasant, unpleasant and neutral ongoing stimuli were pseudo-randomly presented, whereas the valence of the PM cues was kept constant. The order of the three PM sessions was counterbalanced across participants. Each of the PM sessions was divided in 5 blocks of 55 ongoing stimuli and 5 PM cues each, for a total of 300 stimuli per session. In the course of a PM session, 72 one-back hits (24% of 300 stimuli) were presented. PM cues were never also one-back hit trials.

Each block was preceded by an encoding phase, during which five PM cues were presented, one after the other, in the center of the screen for 2000 ms, followed by 1000 ms of blank screen. Before the PM sessions, participants were given 39 ongoing trials of practice (13 trials per valence). At the end of the PM sessions, to test participants retrospective memory for the PM cues, participants were given a recognition task, in which they were asked to recognize the 75 PM cues (25 PM cues for each of the three PM sessions). Seventy-five filler pictures taken from the ongoing task served as distractors. Here, participants responded by pressing one of two possible keys with the index or middle finger of their right hand (“T” or “Y” keys on the keyboard). Again, the response-key mapping was counterbalanced across participants.

### Behavioral analysis

All dependent variables considered were subjected to repeated-measures analyses of variance (ANOVA), with a probability of a type I error being set to 0.05. We estimated effect sizes using partial eta squared (η^2^_*p*_). *Post hoc* comparisons were carried out using Newman-Keuls tests.

In order to investigate the effect of emotional valence of the PM cue on the ongoing task performance, two ANOVAs were run on the RTs and percentage of accuracy in ongoing task with two within-subjects factors: Valence of the PM session (unpleasant, neutral, pleasant) and valence of the ongoing stimuli (unpleasant, neutral, pleasant). An ANOVA with the valence of the PM cue as factor was applied to the percentage of accuracy in the PM task. Finally, to investigate the effect of PM cue valence on the recognition task, RTs in the recognition task were analyzed by means of two 2 × 3 ANOVAs, with stimulus type (ongoing vs. PM) and valence of the stimuli (unpleasant, neutral, pleasant) as within-subjects factors.

Notably, we examined accuracy on recognition task within the Stimulus Detection Theory (SDT) framework and tested whether valence of PM cue led to changes in recognition and/or to decision/response bias (Stanislaw and Todorov, 1999). The SDT framework provides a simple method to disentangle retrieval and decision aspects of recognition memory (Verde and Rotello, 2007; for a review, see Clark and Gronlund, 1996). We recorded hits (PM cues called “PM cues”) and false alarms (ongoing trials called “PM cues”). To compute the signal detection values *d*′ and β, hit and false alarm proportions were corrected according to the log-linear rule (e.g., Hautus, 1995). In the present study, the *d*′ value provides information about the ability to discriminate between PM cues and ongoing trials, whereas β reflects a potential bias of individuals to respond “PM cue” even when the ongoing trials were presented (or *vice versa*). Finally, both *d*′ and β values were entered into an ANOVA, with valence of the stimuli as independent variable.

### Electrophysiological recording and analysis

EEG was recorded (EEG equipment: System Plus, Italy) from an array of Ag/AgCl scalp electrodes mounted on an elastic cap (ElectroCap International, Inc.) and positioned according to the 10–20 International System. The montage included the following scalp positions: Fp1, Fpz, Fp2, AFz, F7, F3, Fz, F4, F8, Fc3, Fcz, Fc4, T3, C3, Cz, C4, FT7, FT8, T3, T8, T5, Cp3, Cpz, Cp4, P3, PZ, P4, T6, Tp7, Tp8, O1, O2, and Right and Left Mastoids. Eye movements were monitored by two electrodes using a bipolar montage, with one electrode placed below the right eye, and one placed on the external canthi of the left eye. The ground electrode was placed in AFz. Data were digitized at a sampling rate of 512 Hz. Electrode impedance was kept below 5 kΩ. Data processing was performed with EEGLAB 13.1.1b (Delorme and Makeig, 2004), running in a Matlab environment (Version 7.9.0, MathWorks, Natick, MA, USA). Continuous EEG was filtered between 0.1 and 30 Hz and resampled at 256 Hz. Then, it was segmented into epochs starting 200 ms prior to and ending 2400 ms after stimulus presentation. Artifact correction was done on these epochs by means of the Independent Component Analysis (ICA) toolbox in EEGLAB. Only epochs with correct responses were included in the analysis. Afterwards, epochs were re-segmented, including 200 ms of pre-stimulus and 1400 ms post-stimulus activity. Baseline correction was made using the 200 ms pre-stimulus activity. Epoch rejection was performed with a threshold of ±75 μV. However, for most of the conditions and participants, none of the epochs needed to be excluded based on this threshold, suggesting that the signals were, in general, very good. All electrodes were re-referenced offline to the average of the left and right mastoids.

### Partial least squares analysis

To examine the effects of emotions on the ERP correlates of PM, we used the Partial Least Squares (PLS) analysis (Lobaugh et al., 2001; McIntosh and Lobaugh, 2004). PLS analysis is a multivariate statistical technique that can identify differences in ERP amplitude between experimental conditions across time and space, taking into account the full time course and topography of the scalp. Thus, it is a data-driven approach. The ERP input data matrices for the PLS analyses contained participants and conditions in the rows, and ERP amplitudes for all time points and channels, except for the 2 ocular electrodes, in the columns. Analyses were restricted to the post-stimulus interval, from 0 to 1400 ms. The analyses were conducted with the ERP module of the PLSGUI (http://www.rotman-baycrest.on.ca) implemented in Matlab. The significance of the latent variables (LVs) values was calculated using a permutation test (1000 replications). An LV was considered significant at *p* < 0.05. To prevent the effects of possible outliers, the stability of the ERP saliences in space and time was established through bootstrap resampling (200 replications) that provides a standard error. Bootstrap ratios > 3 were chosen as the cut-off for stable non-zero saliences.

To analyse ERP effects across the four process phases of our paradigm, four separate PLS analyses were conducted:

1. The first analysis considered the ERPs elicited by unpleasant, neutral and pleasant pictures in the *encoding* phase. Note that given the low number of misses in the PM task, we could not investigate the ERPs at the encoding phase comparing PM hits with PM misses.
2. The second analysis targeted the *maintenance* phase and considered the ERPs elicited by ongoing stimuli and included the 9 task conditions obtained by crossing 3 levels of PM session valence (unpleasant, neutral, pleasant) and 3 levels of ongoing stimuli valence (unpleasant, neutral, pleasant).
3. The third analysis was done on the *retrieval* phase and considered the ERPs elicited both by the ongoing stimuli and the PM cues. To balance the conditions in the experimental analysis, we compared the ERP signal of the PM cues with the ongoing stimuli having the same valence of the PM session. Thus, the analysis included 6 task conditions: ongoing unpleasant, neutral and pleasant stimuli and PM unpleasant, neutral and pleasant cues.
4. The fourth analysis targeting the retrospective component of PM was done on the *recognition* phase and considered the ERPs elicited by stimuli in the recognition task and included 6 task conditions obtained by crossing 2 levels of stimulus type (ongoing, PM cue) and 3 levels of valence (unpleasant, neutral, pleasant).

## Results

### Behavioral results

The behavioral mean values are displayed in Table 1. The analysis of RTs in the ongoing task revealed a significant main effect of valence of the ongoing stimuli [*F*_(2, 46)_ = 10.92, *p* < 0.05, η^2^_*p*_ = 0.32]. RTs were slower for unpleasant stimuli compared to both pleasant and neutral stimuli (*ps* < 0.05). Furthermore, RTs tended to be slower for pleasant than for neutral stimuli (*p* = 0.6). The interaction between the valence of the PM session and the valence of the ongoing stimuli was significant [*F*_(4, 92)_ = 17.29, *p* < 0.05, η^2^_*p*_ = 0.43], revealing the presence of the SSIE (Stimulus Specific Interference Effect). Indeed, *post hoc* comparisons showed that, in the unpleasant PM session, RTs were slowest for unpleasant pictures, intermediate for pleasant pictures, and fastest for neutral pictures (all *ps* < 0.05). In the pleasant PM session, RTs were slowest for pleasant pictures, intermediate for unpleasant pictures, and fastest for neutral pictures. In the neutral PM session, RTs were slower for neutral and unpleasant pictures than for pleasant pictures (all *ps* < 0.05).

**Table 1:** **Behavioral results in the Ongoing, PM and Recognition task**.

	**Valence of pictures**		
	**Unpleasant**	**Neutral**	**Pleasant**
**ONGOING TASK**			
**Mean RTs (SD)**			
***PM session***			
Unpleasant	647 (107)	601 (83)	620 (97)
Neutral	616 (98)	620 (113)	595 (91)
Pleasant	625 (121)	603 (112)	636 (128)
**ONGOING TASK**			
**% Accuracy (SD)**			
***PM session***			
Unpleasant	96.7 (2.5)	96.7 (2.7)	96.5 (2.1)
Neutral	96.7 (1.6)	96.8 (2.8)	97.6 (2.3)
Pleasant	96.9 (2.6)	96.4 (6.4)	96.9 (3.4)
**PM TASK**			
**% Accuracy (SD)**			
PM cue	95.7 (4.9)	92.1 (9.8)	93.7 (6.4)
**RECOGNITION TASK**			
**Mean RTs (SD)**			
***Stimulus type***			
Ongoing	734 (81)	728 (100)	754 (100)
PM cue	824 (99)	821 (109)	850 (91)
**RECOGNITION TASK**			
**% Accuracy (SD)**			
***Stimulus type***			
Ongoing	92.3 (5.5)	95.6 (4.2)	95.8 (6.4)
PM cue	77.4 (1.7)	76.8 (1.6)	72.6 (2.0)

No significant effects were found in terms of accuracy on the ongoing task (all *ps* > 0.05). Accuracy in PM performance was high for all the emotional valence conditions (see Table 1) and was not significantly influenced by PM cue valence [*F*_(2, 46)_ = 1.68; *p* > 0.05; η^2^_*p*_ = 0.08].

In the recognition task, there was a significant main effect of stimulus type on RTs [*F*_(1, 23)_ = 27.10, *p* < 0.05, η^2^_*p*_ = 0.54], with RTs being faster for the ongoing stimuli than for the PM cues (Table 1). The effect of valence was also significant [*F*_(2, 46)_ = 5.52, *p* < 0.05, η^2^_*p*_ = 0.19]. Pleasant pictures were indeed characterized by slower RTs compared to both unpleasant and neutral pictures, regardless of stimulus type (*ps* < 0.05). The interaction between stimulus type and valence of the picture was not significant (*p* > 0.05).

We analyzed recognition performance in two steps, considering the effects of stimulus valence on the ability of discriminability (*d*′ value) and on response bias (β value, respectively. The *d*′ values did not significantly vary across the unpleasant (*d*′ *mean* = 2.22; *SD* = 0.83), neutral (*d*′ *mean* = 2.39; *SD* = 0.70), and pleasant (*d*′ *mean* = 2.32; *SD* = 0.75) stimuli [*F*_(2, 46)_ = 0.50, *p* > 0.05], suggesting that valence of stimuli did not significantly affect individuals' ability to recognize PM cues. On the other hand, the β value significantly differed as a function of stimulus valence [*F*_(2, 46)_ = 3.69, *p* < 0.05, η^2^_*p*_ = 0.14], with unpleasant stimuli being characterized by smaller β (β *mean* = 4.88; *SD* = 6.28) as compared to both neutral (β*mean* = 9.61; *SD* = 5.93) and pleasant (β*mean* = 9.30; *SD* = 9.24) stimuli (*ps* < 0.05). This pattern of results indicates that participants tended to more often classify the ongoing stimuli as “PM cues” when such stimuli were unpleasant (rather than neutral or pleasant).

### ERP results

**Figure 1:**
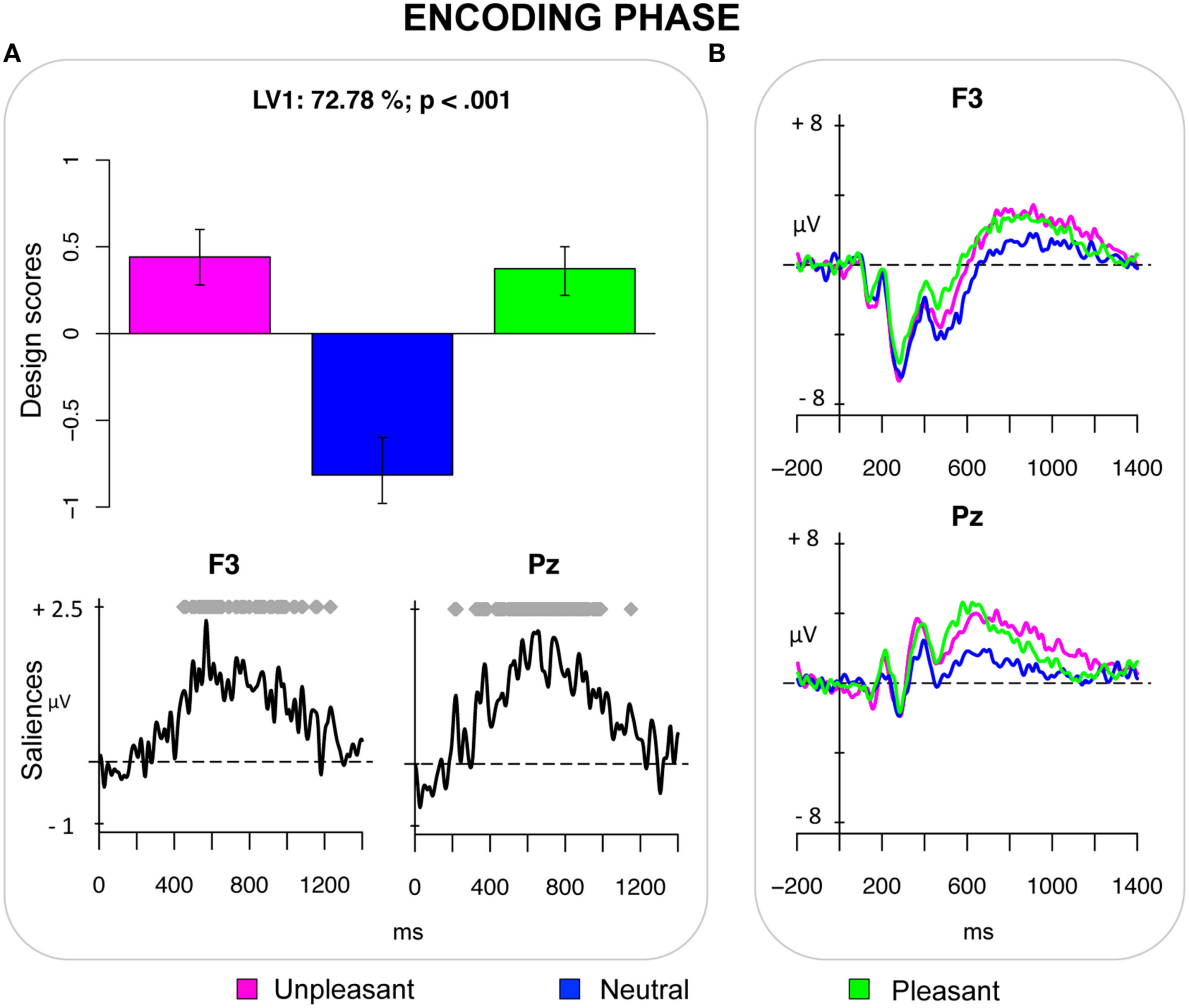
**Encoding phase. (A)** Design scores demonstrating a contrast between emotional pictures and neutral pictures and saliences at select electrodes. **(B)** Grand-averaged event-related potentials (ERPs) at select electrodes showing an increased sustained positivity for emotional pictures in the time window between 400 and 1000 ms after stimulus onset. This positivity is widely distributed over the scalp, being expressed from frontal electrodes (especially the left ones), to the parietal electrodes.

The first PLS analysis (*encoding phase*) included ERPs elicited by unpleasant, neutral and pleasant pictures in the encoding phase. The analysis revealed one significant LV (*p* < 0.001), which accounted for 72.78% of the crossblock covariance and reflected a contrast between emotional pictures (both pleasant and unpleasant) and neutral pictures. It captured the effect of valence of the PM cue, which was expressed as a sustained positivity over parietal and centro-parietal electrodes and over left frontal electrodes in the time window roughly between 400 and 1000 ms post-stimulus (Figure 1).

**Figure 2:**
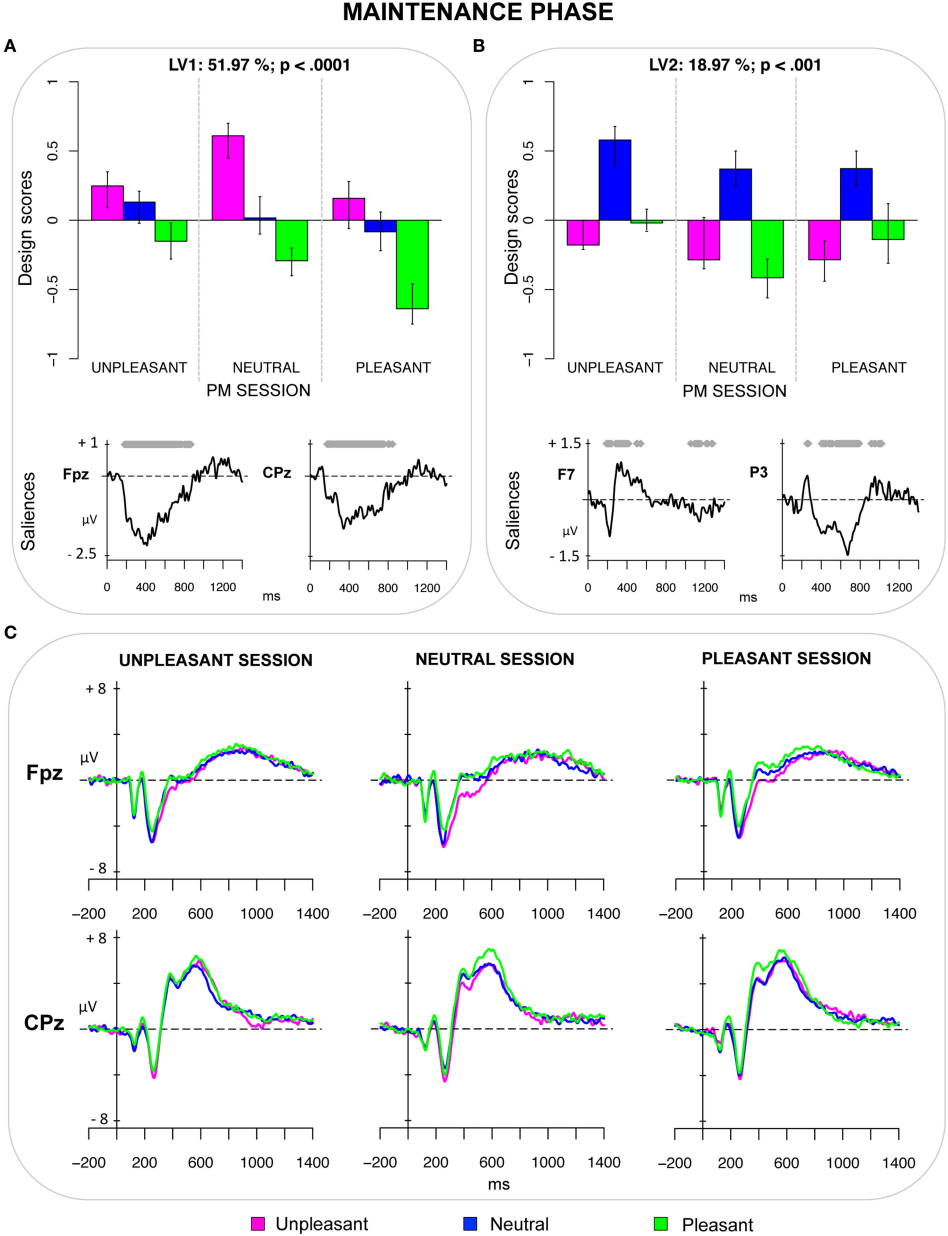
**Maintenance phase. (A)** Design scores and saliences at select electrodes for LV1, contrasting pleasant from unpleasant pictures in ongoing task. LV1 reflected an enhanced positivity for pleasant pictures, which was modulated by the valence of the PM cue and was greatest when the PM cue was pleasant. **(B)** Design scores and saliences at select electrodes for LV2, contrasting neutral pictures from emotional pictures in ongoing task. **(C)** The grand-averaged ERPs at select electrodes for the ongoing pictures in the distinct PM sessions.

The second PLS analysis (*maintenance phase*) was applied to the ERPs elicited by unpleasant, neutral, and pleasant ongoing trials in the three PM sessions (unpleasant, neutral, and pleasant). The analysis revealed two significant LVs (both *ps* < 0.001) that accounted for 51.97 and 18.32% of the cross-block covariance, respectively (see Figure 2). LV1 represented a contrast between pleasant and unpleasant pictures in the three PM sessions (Figure 2A). Moreover, the magnitude of the design scores for these pictures was modulated by the valence of the PM session. It is noteworthy that the design scores for pleasant pictures were greatest (i.e., more negative) in the pleasant PM session, intermediate in the neutral session and smallest in the unpleasant PM session. Thus, for the pleasant pictures, the effect of emotional valence interacted with monitoring processes recruited to detect the PM cues. The magnitude of the design scores for unpleasant pictures also differed depending on the valence of the PM session, but the pattern of results was less clear in this case.

The bootstrap test revealed a sustained modulation that was different from zero in the epoch starting roughly from 160 ms and ending at 900 ms, and that was expressed over the scalp from the prefrontal to the centro-parietal regions. This modulation reflected an increased widespread positivity for pleasant pictures compared to the other pictures, especially the unpleasant ones. As can be seen from Figure 2C, such a positivity elicited by pleasant pictures was modulated by the valence of the PM session, being lowest in the unpleasant session, intermediate in the neutral session and highest in the pleasant session (see also Supplementary Materials for an in-depth exporation of this pattern of results).

The LV2 distinguished between emotional (unpleasant and pleasant) pictures and neutral ones (Figure 2B). This LV reflected, over lateral frontal regions, two transient modulations within early time windows (i.e., 170–250 ms and 280–430 ms), which represented an increased positivity followed by an increased negativity for emotional ongoing pictures (regardless of PM session), respectively. Over posterior regions (especially over the lateral parietal and occipital sites), the LV reflected an increased sustained positivity, for the emotional pictures, in the time-window of 300–800 ms.

**Figure 3:**
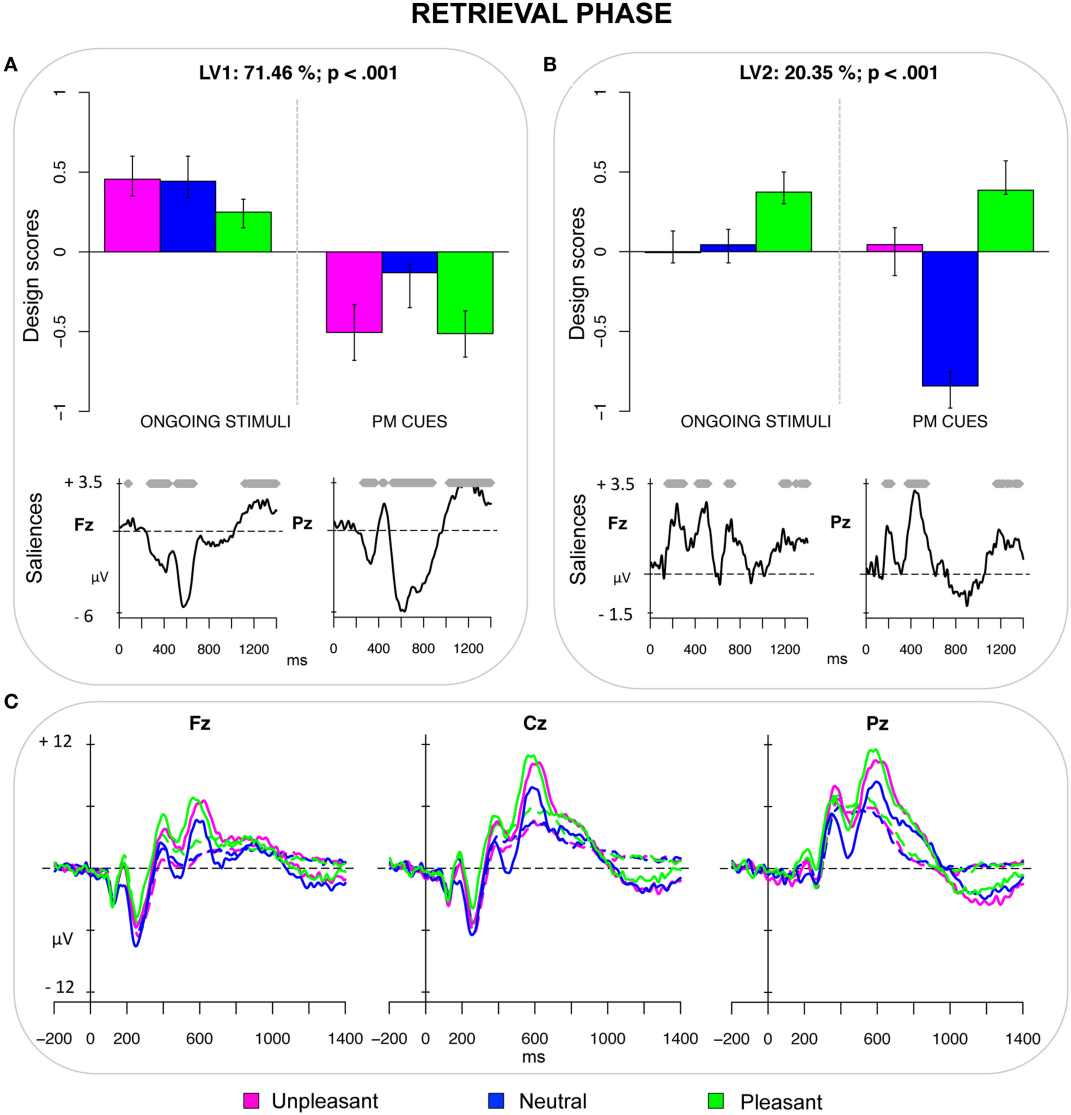
**Retrieval phase. (A)** Design scores and saliences at select electrodes for LV1, contrasting emotional PM cues from ongoing stimuli. The electrode saliences represented a frontal positivity and a parietal positivity, which was the result of the prospective positivity and the LPP. **(B)** Design scores and saliences at select electrodes for LV2, distinguishing pleasant pictures from neutral PM cues. The electrode saliences captured an increased widespread positivity for pleasant pictures in both earlier and later time windows. **(C)** The grand-averaged ERPs at midline electrodes for ongoing pictures (dotted lines) and PM cues (solid lines) as a function of the emotional valence.

The third PLS analysis (*retrieval phase*) included the ERPs elicited by the PM cues and by the ongoing stimuli having the same emotional valence of the PM cues in that session. This analysis revealed two significant LVs (both *ps* < 0.001) that accounted for 71.46 and 20.35% of the crossblock covariance, respectively. LV1 reflected a contrast between ongoing stimuli and PM cues, but especially emotional PM cues (i.e., pleasant and unpleasant) as can be seen in Figure 3A. Thus, such LV captured a pattern of ERPs reflecting the interplay between PM processes and emotion-related processes. LV1 represented a series of modulations in the time window between 250 and 900 ms. More specifically, emotional PM cues were characterized by a positive peak at about 400 ms, particularly pronounced over frontal regions, followed by a relative negative deflection especially over occipital sites (peaking at 460 ms) and a large sustained positivity mainly expressed over central and parietal sites (starting roughly at 500 ms), which might reflect the result of two overlapping deflections: the prospective positivity and the LPP. Even though these modulations were widespread over the scalp, they were more pronounced over central and parietal regions (Figure 3C).

LV2 represented a contrast between pleasant pictures (both the PM cues and ongoing stimuli) and the neutral PM cues (Figure 3B). The bootstrap test showed that the electrode saliences were different from zero in two temporal windows (150–250 and 360–550 ms) that reflected increased widespread positivity for pleasant stimuli, occurring over frontal, central and parietal sites of the scalp.

**Figure 4:**
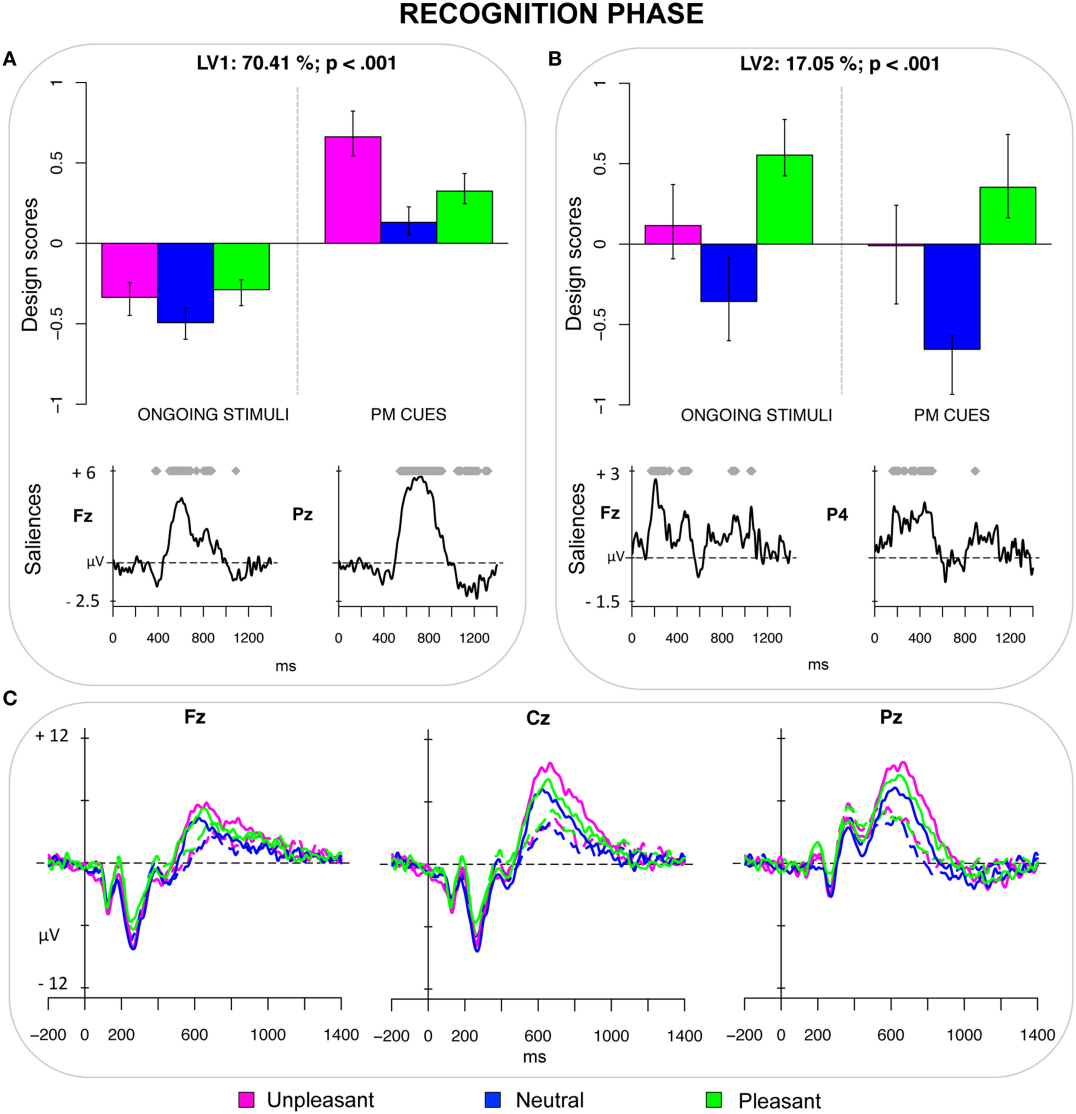
**Recognition phase. (A)** Design scores and saliences at select electrodes for LV1, contrasting emotional recognized PM cues (especially unpleasant ones) from recognized ongoing stimuli. The electrode saliences represented a diminished frontal positivity (i.e., the FN400) and an increased parietal positivity (old/new effect and LPP) for the recognized PM cues. **(B)** Design scores and saliences at select electrodes for LV2, distinguishing pleasant pictures from neutral PM cues. The electrode saliences captured an increased positivity for pleasant pictures, which was widely expressed over the scalp but more pronounced over the right hemisphere. **(C)** The grand-averaged ERPs at midline electrodes for recognized ongoing pictures (dotted lines) and recognized PM cues (solid lines) as a function of the emotional valence.

The fourth PLS analysis (*recognition phase*) included the ERPs elicited by ongoing stimuli and PM cues included in the recognition task. This analysis revealed two significant LVs (both *ps* < 0.001) that accounted for 70.41 and 17.05% of the crossblock covariance, respectively. LV1 distinguished the recognized PM cues (especially the emotional PM cues) from the recognized ongoing pictures (Figure 4A). It is noteworthy that the higher design score was obtained for unpleasant PM cues. Thus, this LV seems to represent the interaction between the recognition effect and the emotion effect. The recognition effect was modulated by the emotional valence and was expressed in two time windows. The first modulation represented a transient increased frontal positivity, for the ongoing pictures, which occurred in the 300–400 ms time window and was likely to reflect a FN400. The latter modulation reflected an increased sustained positivity for the emotional PM cues (especially for the unpleasant ones), in the time window between 500 and 900 ms. This modulation was widely expressed over the scalp, but was particularly pronounced over the parietal sites, and was the result of the overlap between the recognition old/new effect and the LPP (Figure 4C).

LV2 distinguished between pleasant and neutral pictures, regardless of the type of the stimulus (ongoing vs. PM). The ERP modulations captured by LV2 were expressed as increased positivity for pleasant stimuli in the time windows between 150–300 ms and 400–500 ms. These modulations were widely distributed over the scalp (from frontopolar to parietal sites), especially over the right hemisphere.

## Discussion

### Emotion effects on behavioral measures of PM

The first goal of the present study was to clarify the role of PM cue valence on behavioral measures related to PM performance. Examination of the RTs for the ongoing task revealed the SSIE (Stimulus Specific Interference Effect; Marsh et al., 2003; Cohen et al., 2012; Scolaro et al., 2014), namely, an increase in RTs for the ongoing pictures that had the same emotional valence of the PM cue. This finding suggests that attentional resources recruited for target checking were greatest when the emotional valence of the ongoing pictures matched that of the current PM cue. Interestingly, a smaller but significant interference effect in the RTs was also observed for the ongoing pictures that had the opposite valence of the PM cue. This suggests that ongoing pictures having an emotional valence that was opposite to the one of the PM cue were monitored for as well, possibly because their emotional and arousing nature was linked, even if reversed, to the emotional nature of the PM cue.

The accuracy of PM performance was generally high, and was not significantly affected by valance of PM cue. This was not surprising because, due to the ERP approach of the study, we decided to use an ongoing and PM task that were both not particularly demanding. This choice ensured to have relatively high PM performance, preventing the need for rejecting too many epochs for the PM. Our finding is also in line with the study by Altgassen et al. (2010), which showed that valence of the PM cue had no impact on PM performance in young individuals.

In addition, two novel and conceptually interesting results were revealed by the performance in the recognition task. First, as compared with pleasant and neutral pictures, unpleasant pictures tended to more often be judged as PM cues even if they were ongoing stimuli, as revealed by the smaller β value for unpleasant stimuli than for neutral and pleasant stimuli. This bias toward PM cues, selectively for unpleasant stimuli, parallels the “emotion-induced recognition bias effect,” that reflects the individuals' tendency to respond “old” more likely to unpleasant stimuli than to neutral stimuli, independent of whether the stimulus is actually new or old (Windmann and Krüger, 1998; Windmann and Kutas, 2001). This bias has been considered the result of an adaptive cognitive mechanism built in to guarantee that information or events with a potentially high survival meaning are not forgotten or ignored (Windmann and Krüger, 1998). Second, evaluating pleasant pictures (regardless of whether they were PM cues or ongoing stimuli) seems to take more time compared to evaluating unpleasant and neutral pictures. This might be due to the fact that individuals do not invest much effort in processing and consolidating pleasant information, possibly because of overestimating their ability to recall them (Murphy and Isaacowitz, 2008).

### Emotion effects on ERPs

The second, and more important, goal of the present study was to target possible mechanisms of emotion–PM interactions and therefore, for the first time, disentangle the effects on a neural level at the various phases of PM, from the initial encoding via maintaining of PM intentions to their retrieval, execution and recognition. To accomplish this aim, and to highlight the neural correlates of such emotion–PM interactions, we explored the ERPs elicited in the distinct phases.

### Encoding phase

The first PLS analysis on the ERPs revealed that emotional pictures were differentiated from the neutral pictures already in the encoding phase. As compared with neutral pictures, both pleasant and unpleasant pictures elicited a more positive-going ERP modulation—the LPP—that was expressed over central and parietal regions and over left frontal regions roughly between 400 and 1000 ms post-stimulus (Figure 1). Given that the LPP is affected by attention and is related to stimulus evaluation, such LPP modulations suggest an increase in attentional processes recruited for evaluating and encoding the emotional stimuli (Hajcak et al., 2006, 2009; Moser et al., 2006; Weinberg and Hajcak, 2010). This finding is consistent with the study by Dolcos and Cabeza (2002) on memory formation, which reported that the effect of emotion on ERPs was similar for pleasant and unpleasant to-be-encoded stimuli, thus suggesting that arousal could have a key role in this phase (see also May et al., 2012, 2014). As compared with their study, however, the present study found that the emotion effect on ERPs was more long-lasting and that it was lateralized over left frontal regions for both pleasant and unpleasant pictures. Despite the low spatial resolution of the ERPs, we assume that left frontal regions might contribute in mediating the encoding of intention, as recently demonstrated by a meta-analysis of neuroimaging studies of PM (Cona et al., under review; see also Poppenk et al., 2010). Clearly, future studies will have to elaborate these tentative conclusions using other neuroscience methodologies allowing for higher spatial resolution.

### Maintenance phase

The second PLS analysis was performed on the ERPs elicited by the ongoing stimuli in order to explore the effect of emotion on the processes recruited during the maintenance phase, such as strategic monitoring. The LV1 captured a contrast specifically between pleasant and unpleasant pictures, with pleasant pictures being characterized by a greater positivity in the time window starting roughly at 160 ms and ending at 900 ms (Figure 2). This effect was widespread over the electrodes of the scalp, but more prominent over prefrontal electrodes.

Notably, the magnitude of the design scores for the emotional pictures differed across the sessions as a function of the valence of the PM cue, thus revealing emotion–PM interactions in this phase. Particularly, the sustained positivity elicited by pleasant ongoing pictures was greatest when the PM cues were pleasant, too, intermediate when the PM cues were neutral, and smallest when they were unpleasant. The pattern of ERPs captured by the LV1 could be seen as reflecting, at least in part, a SSIE, in which monitoring is boosted by the match of valence of the ongoing stimulus with the valence of the PM cue (see also Supplementary Materials for an in-depth exploration and speculation of this pattern of results). In this case, for instance, ongoing pleasant pictures showed greater positivity in the session with pleasant PM cues (than in sessions with unpleasant and neutral PM cues). This *post hoc* hypothesis is also driven by previous studies, which found that the ERP correlates of target checking were sensitive to the defining attributes of the PM cues (Knight et al., 2010; Cona et al., 2014; Scolaro et al., 2014). The LPP might contribute to elicit such an enhanced positivity as well, as suggested by the temporal dynamic and spatial distribution of the modulations represented by the LV1. This would be in line with previous reports of an amplification of the LPP when the emotional content is relevant for the task (Schupp et al., 2000; Hajcak et al., 2009). In the PM context, such an enhancement is likely to reflect the allocation of increased monitoring resources toward pleasant ongoing pictures when these pictures are more relevant for the PM task, as when they match the PM cue in terms of valence (Schupp et al., 2000, 2003; Hajcak et al., 2009).

Nevertheless, this pattern of results leads to a further question: Why was the SSIE especially reflected in the ERP modulations of pleasant pictures? The ERPs elicited by the unpleasant ongoing stimuli were modulated by the valence of the PM session as well, but their pattern of ERP modulations was less clear. One potential hypothesis is that the allocation of attention toward unpleasant stimuli was somehow more constant across the PM sessions. This interpretation is in line with the pattern of RTs on ongoing trials, which revealed that, while RTs for pleasant ongoing stimuli differed greatly depending on the PM session, RTs for unpleasant ongoing stimuli were less affected by the PM session and were in general slower when compared with RTs for pleasant and neutral ongoing stimuli. A possible explanation is related to the “negativity bias” framework, which emphasizes the intrinsic relevance of threatening and unpleasant stimuli (Öhman and Mineka, 2001; Crawford and Cacioppo, 2002). According to this framework, individuals would have a predisposition to allocate and maintain attention toward threatening and unpleasant stimuli in order to facilitate processing efficacy (Olofsson et al., 2008).

The LV2 derived from the PLS analysis on ongoing pictures captured the effect of emotion, distinguishing between emotional pictures and neutral pictures. The emotion effect was expressed over frontal electrodes as an early transient increased positivity followed by a transient increased negativity for emotional pictures (regardless of PM session). Over posterior sites, it was represented by an increased sustained positivity between 300 and 800 ms, namely the LPP. In line with the conclusions suggested in previous studies, these modulations seem to reflect the obligatory attentional capture (the earlier modulation) as well as the holding of top-down attention toward emotional stimuli (the later modulation) (e.g., Azizian and Polich, 2007; Olofsson et al., 2008; Foti et al., 2009; May et al., 2012).

### Retrieval phase

The LV1 of the third PLS analysis distinguished emotional PM cues (whereas, only to a lesser degree, the neutral PM cues) from ongoing stimuli, thus revealing an interplay between emotion and PM processes in the retrieval phase (Figure 3). This might be the result of the enhanced processing of emotional PM cues in the encoding phase, which led to a better differentiation of such PM cues amid the ongoing stimuli at retrieval. More specifically, emotional PM cues were found to elicit two positive components: the frontal positivity, which occurred between 300 and 450 ms after stimulus presentation; and the parietal positivity, which occurred between 500 and 900 ms. The early frontal positivity has been typically related to more automatic processes, such as involuntary capture of attention (i.e., the P3a; Delplanque et al., 2006; Cona et al., 2013) and spontaneous recognition of the stimulus (i.e., the FN400; Jennings and Jacoby, 1993; Curran, 2000). Based on such findings, one could speculate that emotional PM cues might boost automatic processes for detecting the PM cue. This explanation is corroborated by recent studies, showing that emotion increases the saliency or distinctiveness of a PM cue, thereby improving its detection (May et al., 2012, 2014). The PM cues elicited also a parietal positivity, which is the product of multiple overlapping components and thus reflects multiple processes such as the contextual updating (P3b), the retrieval of the intention from mind (the old/new effect), and the coordination between PM and ongoing actions (*prospective positivity*; West and Wymbs, 2004; Bisiacchi et al., 2009; West, 2011). The emotion-related enhancement of the parietal positivity would suggest an increased allocation of resources to accomplish these processes when the PM cues were emotional. This is in agreement with studies showing that, as compared with neutral stimuli, emotional stimuli tend to receive increased processing (e.g., Weinberg and Hajcak, 2010). Motivational factors have been proposed to account for the enhancement in the allocation of attention toward emotional stimuli (e.g., Lang et al., 1997; Sabatinelli et al., 2005), thus it is possible that they played a role also in the present study. However, future studies will be required to test this hypothesis within the PM context.

Taken together, the results from the third PLS analysis suggest that emotional material, on one hand tended to stimulate bottom-up and spontaneous processes to detect the PM cues, on the other hand encouraged to recruit and hold top-down controlled resources to retrieve and execute the associated intention. Notably, since this difference has been reflected only in the design scores of the PM cues, this pattern does not concern the effect of emotions in general but is specific to the PM cues.

### Recognition phase

The interaction between emotion and PM functioning was revealed also in the recognition phase (Figure 4). The LV1 of the PLS analysis on the ERPs elicited in the recognition task distinguished the correctly recognized emotional PM cues from the correctly recognized ongoing task stimuli. Notably, this distinction was amplified for unpleasant PM cues, which had the greatest design scores. The recognized emotional PM cues (and especially the unpleasant PM cues) were characterized by a transient decreased frontal and central positivity in the 300–400 ms time window, and by an increased sustained positivity in the time window between 500 and 900 ms. Such later modulation was widely expressed over the scalp, but was particularly pronounced over the parietal sites, and was likely to be the result of the overlap between the recognition old/new effect and the LPP. The correct classification of the ongoing pictures or, in other words, rejecting that these stimuli were PM cues seemed to be mediated by bottom-up and automatic processes. This conclusion is motivated by the observation that recognized ongoing stimuli were characterized by an enhanced positivity in the time window of the FN400, a component linked to automatic aspects of memory recollection (e.g., Jennings and Jacoby, 1993; Curran, 2000; Cona et al., 2014), and by faster and more accurate responses compared to recognized PM cues. Based on these results, it seems plausible to conclude that individuals found it easier and were faster to reject the ongoing pictures, rather than to identify the PM cues.

The old/new effect was increased at 500–900 ms for emotional PM pictures, and was more pronounced for the unpleasant ones than for neutral and pleasant PM pictures, replicating previous studies in the episodic memory literature (Dietrich et al., 2001; Johansson et al., 2004; Inaba et al., 2007; Weymar et al., 2009). Since the old/new effect is thought to reflect conscious and strategic recollection of memory contents (Allan et al., 1998; Rugg et al., 1998), the larger old/new effect for emotional PM cues suggests that an increased allocation of strategic resources subserves the recognition of emotional stimuli as “PM cues.” Notably, observing the largest old/new effect for unpleasant PM cues is consistent with the findings of Johansson et al. (2004) and Ochsner (2000). It mirrors the “emotion-induced recognition bias effect” and suggests that individuals tend to overestimate the number of unpleasant PM cues, likely because unpleasant information attains a privileged status in memory, especially when it is relevant, as in the case of PM cues (Cacioppo et al., 1999).

Finally, the LV2 of both the third (*retrieval phase*) and fourth (*recognition phase*) PLS analysis captured a difference between pleasant and neutral pictures, regardless of the type of stimulus (i.e., ongoing vs. PM) and revealed similar electrode saliences. They seem to reflect a specific effect of pleasant pictures, which lead to an increased positivity in the time windows roughly between 150–300 ms and 400–500 ms post-stimulus. Thus, modulation was widely distributed over the scalp, although it was a little bit more lateralized over the right hemisphere for recognized pictures. As suggested by Bailey et al. (2012), the inclusion of erotic pictures in the sample, as in our study, can account for such larger positivity for pleasant pictures. For the ERPs elicited by the recognition task, this finding might also reflect the greater difficulty in deciding whether the pleasant pictures were ongoing stimuli or PM cues, as it was also observed by the slower RTs for these pictures compared to the other ones.

### Limitations and future directions

There are some limitations of the study that must be taken into account. First, we preferred not to include a baseline block consisting of the ongoing task alone in order avoid fatigue effects due to an overly long duration of the experiment. One might argue that, in doing so, it was not possible to draw specific inferences about strategic monitoring. However, the evidence of the SSIE and monitoring-related ERP modulations revealed that the strategic monitoring was indeed recruited, and that it was stimulated by the match of valence of the ongoing stimuli with the valence of the PM cue. Nevertheless, we acknowledge that future studies using a baseline block will be instrumental in further exploring the interactions between emotions and strategic monitoring on the ERPs. Second, the number of the PM cues that contributed to the ERPs is relatively small, especially if compared with the number of the ongoing stimuli. This is however unavoidable in PM research given that, by definition, a PM cue is a rare event in the context of an ongoing activity. Moreover, we decided to focus mainly on the late components (e.g., the LPP), which are less affected by the number of epochs as compared with the earlier components of the ERPs.

Finally, we did not observe the EPN, which has been associated with processing of emotional stimuli. As discussed by Hajcak et al. (2011) in their chapter on “ERPs and the study of the emotion,” the presence of the EPN depends on the reference selected and is generally revealed with average reference. Nevertheless, in our study we preferred to adopt a mastoid reference given the small number of electrodes used, as also recommended by Hajcak and collaborators.

## Conclusions

Taken together, results seem to have several important conceptual implications as they suggest possible mechanisms of emotion effects on PM functioning by disentangling the effects across PM phases and components. Specifically, they suggest that emotional PM cues both trigger an automatic, bottom-up, capture of attention, and boost a greater recruitment of top-down processes necessary for holding the attention and retrieving the intention from mind. The engagement of these processes might be due to the specific motivational relevance of emotional cues (Hajcak et al., 2009). Furthermore, considering the results in relation to the other predictions derived from the literature, an important and novel finding of the present study is that emotions had an impact on PM already at the encoding phase. Moreover, in terms of associated mechanisms, this effect seemed to be mainly due to the arousing nature of the emotional stimuli given that it emerged for both pleasant and unpleasant conditions.

As expected, we also found emotion-related effects in behavioral and neural correlates of strategic monitoring. Indeed, the pattern of behavioral and ERP results on ongoing task performance revealed that the degree of monitoring allocated toward ongoing pictures was influenced by the similarity of the valence between ongoing stimuli and PM cues.

On the contrary, emotional valence of PM cues did not influence the N300, suggesting that perceptual identification of PM cues was not particularly affected by their emotional content. The recognition of PM cues and the retrieval of the associated intention, instead, seemed to be influenced by emotional valence of PM cues, as revealed by the modulations of the FN400 and of the old/new parietal effect.

Notably, the pattern of ERP results revealed specific effects of pleasant and unpleasant PM cues on PM components. Pleasant pictures modulated especially the prospective component, as revealed by the difference in the amplitude of the ERPs elicited by ongoing stimuli as a function of the relevance of the cue valence for the PM task. Unpleasant pictures modulated especially the retrospective component, as revealed by the largest old/new effect elicited by unpleasant PM cues in the recognition task. Our findings further extend the results of the study by Schnitzspahn et al. (2012), who found that, for older adults, pleasant PM cues had a (positive) impact selectively on the prospective component, whereas unpleasant PM cues had a (positive) impact selectively on the retrospective component.

### Conflict of interest statement

The authors declare that the research was conducted in the absence of any commercial or financial relationships that could be construed as a potential conflict of interest.
