# Supplementary material for: Differential effects of emotional cues on components of prospective memory: an ERP study
Source: Front Hum Neurosci. 2015 Jan 28;9:10. doi: 10.3389/fnhum.2015.00010 (PMC4309118; doi:10.3389/fnhum.2015.00010)
Supplement: Supplementary file 1 [file DataSheet1.PDF]

# *Supplementary Material*

# Differential effects of emotional cues on components of prospective memory: An ERP study

Giorgia Cona[a], Matthias Kliegel[b], & Patrizia S. Bisiacchi[ac*]

a- Department of General Psychology, University of Padua, Via Venezia 8, 35131, Padua, Italy.

b- Department of Psychology, University of Geneva, Boulevard du Pont d'Arve 40, CH-1211 Genève 4. Switzerland.

c- Center for Cognitive Neuroscience, University of Padua, Padua, Italy.

*Corresponding author: Patrizia Bisiacchi; Department of General Psychology – University of Padua; Via Venezia, 8, 35131, Padua, Italy; Phone: +39 049 8276609; Fax: +39 049 827 6600; E-mail: patrizia.bisiacchi@unipd.it

## 1. Supplementary Data

The figures below illustrate the ERPs for each valence category of ongoing stimuli, as a function of the PM session: unpleasant (**red**), neutral (**blue**), and pleasant (**green**) PM session. This representation is somehow 'specular' of that presented in the manuscript.
The gray line highlights the time window identified by the second PLS analysis and represents the electrode salience of the LV1.
As can be seen, the ERPs of pleasant ongoing stimuli differ depending on the PM session. Their amplitude is more positive in pleasant session (green line), thus when there is a match between valence of the ongoing stimuli and valence of the PM session.
The other two figures illustrate the ERPs elicited by unpleasant and neutral ongoing stimuli. For these ongoing stimuli, the ERP differences across the three PM sessions are much reduced. Based on this evidence, we interpreted the pattern captured by the LV1 as reflecting, at least in part, a SSIE (stimulus specific interference effect), in which monitoring is boosted by the match of valence of the ongoing stimulus with the valence of the PM cue. In this case, for instance, pleasant pictures showed greater positivity in the session with a pleasant PM cue (than in sessions with a negative PM cue).

In the second part of this file we added a graph of RTs. In line with this idea, RTs to ongoing stimuli vary as a function of the match of valence of the ongoing stimulus with the valence of the PM cue. It's noteworthy that such differences seem to be greater for pleasant stimuli than for unpleasant and neutral stimuli.
Therefore, we think that the most plausible explanation of the pattern of results captured by the LV1 is that it reflects, at least in part, a SSIE. Such phenomenon is related to both the effect of relevance of the stimulus on the LPP and, from another complementary point of view, the effect of matching on target checking process. However, there are still open issues as, for instance, why valence match leads to greater ERP modulations for pleasant images (compared to the other images).

## 2. Supplementary Figures and Tables

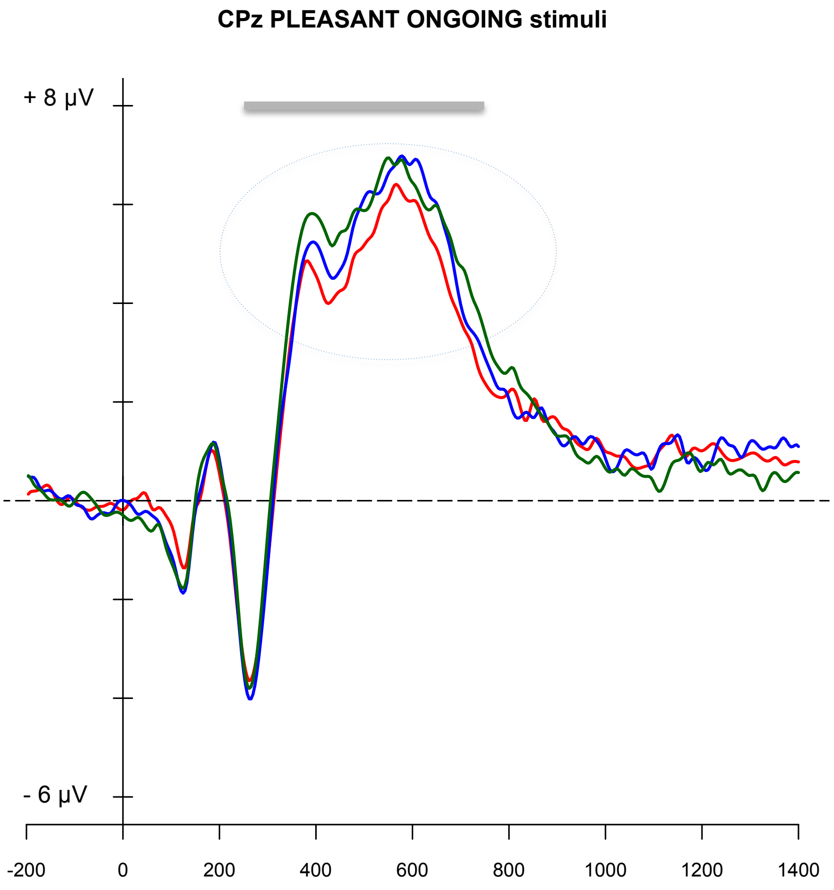


**Supplementary Figure 1. ERPs elicited by pleasant ongoing stimuli in CPz, separately for unpleasant (red), neutral (blue), and pleasant (green) PM sessions.**

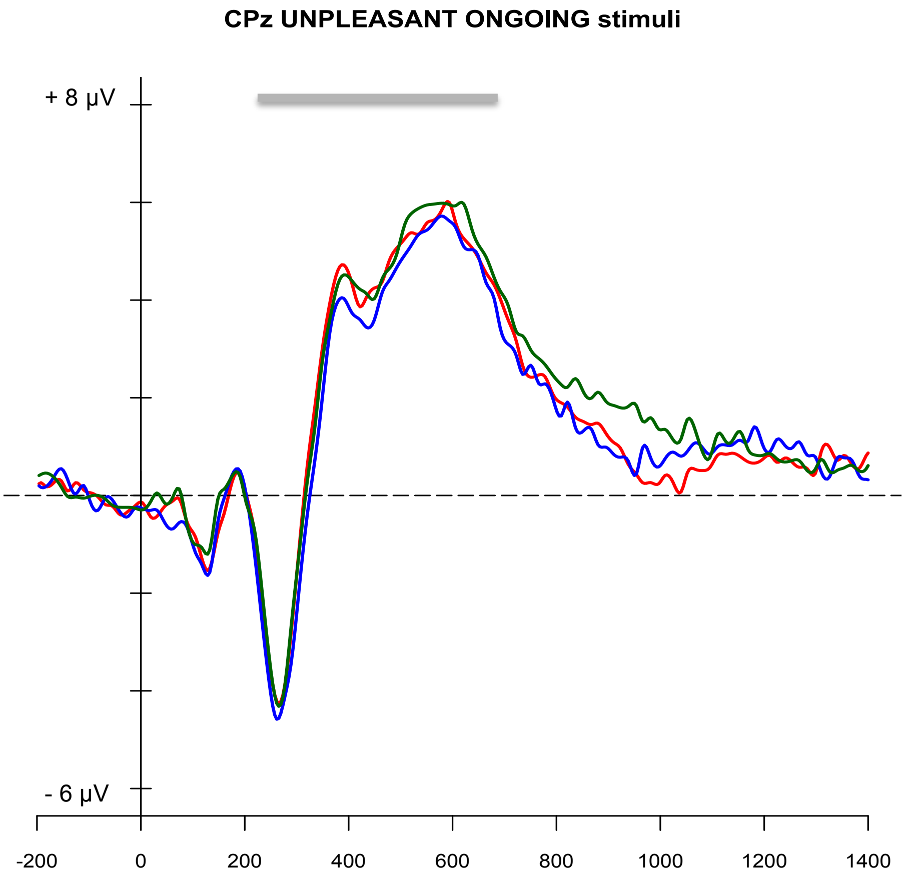


**Supplementary Figure 2. ERPs elicited by unpleasant ongoing stimuli in CPz, separately for unpleasant (red), neutral (blue), and pleasant (green) PM sessions.**

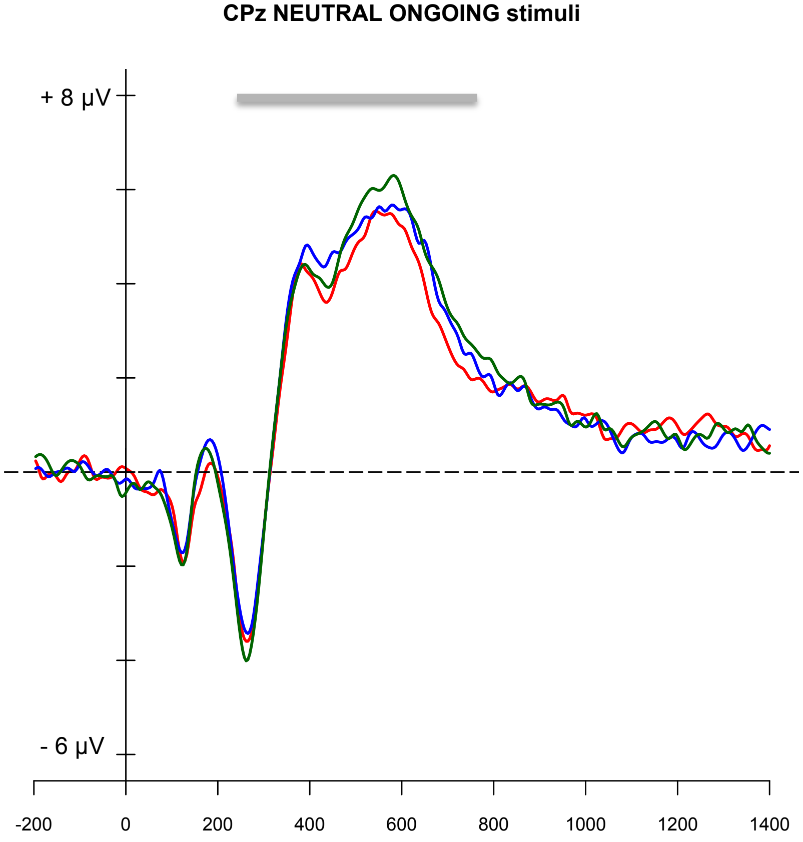


**Supplementary Figure 3. ERPs elicited by neutral ongoing stimuli in CPz, separately for unpleasant (red), neutral (blue), and pleasant (green) PM sessions.**

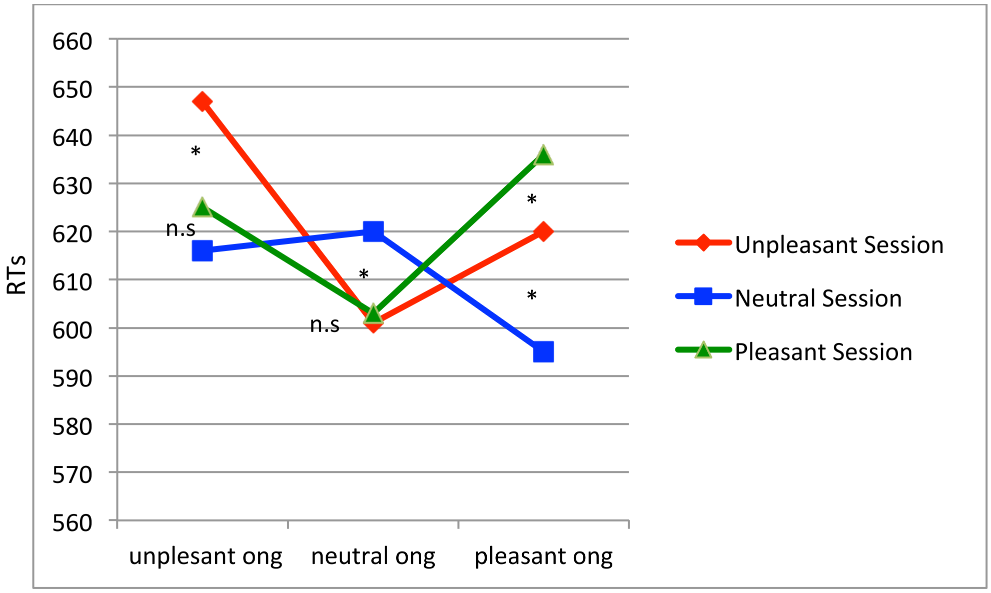


**Supplementary Figure 4. RTs in ongoing stimuli, separately for unpleasant (red), neutral (blue), and pleasant (green) PM sessions.**

**Supplementary Table 1. RTs in the ongoing trials for the three PM sessions.**

| | **unplesant** ongoing stimuli | **neutral** ongoing stimuli | **pleasant** ongoing stimuli |
|---|---|---|---|
| Unpleasant Session | 647 | 601 | 620 |
| Neutral Session | 616 | 620 | 595 |
| Pleasant Session | 625 | 603 | 636 |
| **Greatest RT difference between PM sessions** | **31 ms** | **19 ms** | **41 ms** |
